# Associations of green space visitation patterns with sociodemographics, health, and perceptions: A cluster analysis using smartphone Wi-Fi and GPS data

**DOI:** 10.1371/journal.pone.0325697

**Published:** 2025-06-27

**Authors:** Hwangseon Park, Kwangjune Choi, Changjun Lee, Hayoung Oh

**Affiliations:** 1 Department of Immersive Media Engineering, Sungkyunkwan University, Seoul, South Korea; 2 Department of Applied Artificial Intelligence, Sungkyunkwan University, Seoul, South Korea; Northeastern University (Shenyang China), CHINA

## Abstract

Understanding the multiple impacts of green spaces on individual health and overall quality of life is a key factor in urban planning and public health promotion. This study integrated smartphone Wi-Fi and GPS location data, survey data, and green space data to analyze the relationships between green space visitation patterns and sociodemographic characteristics, health, and green space perceptions of 1,715 residents of the Seoul metropolitan area in South Korea. Green space visitation patterns of urban residents were categorized into Non-Visitors (rarely visited green spaces), Weekday Visitors (weekday visits), Weekend Visitors (weekend visits), and Frequent Visitors (weekday and weekend visits). The health status of residents in each group was assessed using the EQ-5D-5L scale, which evaluates overall mental and physical health. The analysis indicated variations in educational background across groups, with the Non-Visitors and Frequent Visitors showing differing distributions. In addition, the Weekend Visitors group had the best mental and physical health, which were significantly different from the Non-Visitors group. Perceptions of green space were significantly more positive for Weekend Visitors and Frequent Visitors than for Non-Visitors. These results suggest that green space usage patterns can be segmented not only by frequency of visits, but also by when and whether they are visited. It is also worth noting the differences in green space visitation by educational background, highlighting the need for environmental education programs and campaigns to mitigate these environmental inequalities. The positive effect of weekend visits, in particular, highlights the value of green spaces for leisure and relaxation. This finding suggests that urban planning can benefit city residents by providing high-quality, easily accessible green spaces.

## 1 Introduction

Rapid urbanization is gradually reducing the opportunities for human contact with the natural environment [1, 2], and this change can have a negative impact on individual health [3]. As a result, various policies and research are being conducted to create natural environments in urban centers [4, 5]. In particular, green spaces, one of the key elements of natural environments, play an important role in improving the health of urban residents in a variety of ways.

Frequent visits to green spaces have been associated with enhanced well-being and positive emotional states [6, 7]. Green spaces offer psychological benefits such as attention restoration, stress reduction, and emotional regulation [8]. Additionally, green space biodiversity and perceived greenness are both positively linked to mental health [9, 10]. Urban green space exposure is also associated with lower mortality and heart rates, and reduced violence, while encouraging physical activity [11, 12]. These findings demonstrate that the presence of green spaces in residential areas significantly impacts both mental and physical health [13, 14]. Accessibility plays a crucial role in realizing the health benefits of green spaces. When green areas are closer and more abundant, mental health service utilization tends to decrease [3]. For older adults, walkable green spaces are particularly vital, as their presence has been shown to increase survival rates [15]. Interest in the multiple benefits of green spaces has extended to studies analyzing the characteristics of users and the features of frequently visited green spaces [16, 17]. In parallel, there is a growing body of multidisciplinary research focusing on human interaction with green spaces [18]. Recent studies have further explored how urban design and environmental quality influence green space usage. Fine-grained analyses reveal household-level disparities in access, suggesting opportunities to enhance spatial equity by utilizing underused infrastructure such as canals [19].Thermal comfort and microclimate factors such as greenery layout and street orientation significantly affect usability, especially during extreme weather [20, 21]. Urban development patterns also impact ecosystem service value and spatial equity, underlining the need for ecologically sensitive planning [22]. The One Health approach highlights how green space quality, including features like perceived safety and vegetation structure, supports both human well-being and biodiversity [23]. Tree species and landscape design also influence ecological functions like air purification [24]. Cultural ecosystem services (CESs), such as aesthetic enjoyment and spiritual connection, arise from visual elements like trees and flowers [25]. However, green spaces may also lead to disservices such as noise or overcrowding. These dual aspects require planning strategies that balance ecological function with user experience [26].Overall, these findings highlight the need for urban green spaces that holistically address spatial access, environmental quality, ecological function, and user experience to promote well-being and urban sustainability.

Existing studies on human interaction with green spaces have primarily utilized self-report surveys or smartphone-based technologies (e.g., applications, Wi-Fi, GPS, etc.) to capture green space visitation patterns. Self-report surveys have the advantage of being accessible to researchers and useful for capturing qualitative information such as respondents’ perceptions and attitudes. However, they have limitations in objectively identifying actual green space visitation frequency or patterns, as survey responses can be distorted by memory errors, exaggeration, or subjective bias [27, 28]. Overcoming these limitations is critical to understanding the relationships between green space visitation behavior and health and quality of life and requires more objective and reliable data collection methods. To compensate for these limitations, there has been a recent wave of research using smartphone-based technologies (e.g., applications, Wi-Fi, GPS, etc.) to analyze individual activity patterns [29–32]. Smartphones have the advantage of continuously measuring various activities in daily life through multiple built-in sensors and are suitable for human activity recognition research in the health sciences [33]. In particular, attempts have been made to study location accuracy in cities using GPS data from smartphones [34] and to study human mobility data using Wi-Fi and GPS sensors [35], and the reliability of location data based on Wi-Fi and GPS sensors in smartphones is being demonstrated through ongoing research [36–38]. A previous study that analyzed human interaction with green spaces using smartphone-based location data found that users spent most of their time within green spaces on trails, and that there was a significant correlation between human activity and tree composition [39]. In addition, an analysis of green space visitor characteristics found that ethnic minority users and residents of socio-economically disadvantaged neighborhoods tended to spend less time in green spaces compared to white users and residents of more affluent neighborhoods, and that users aged 34 and older spent relatively more time in green spaces than younger users [40]. There is also a growing body of research on why and how individuals choose and utilize urban green spaces, which has implications for urban design and spatial planning by focusing on the interactions between physical environmental elements of green spaces and individual psychological and cultural characteristics [41]. Building on this line of research, recent studies have increasingly leveraged mobile phone and location-based data to gain deeper insights into urban park usage and accessibility patterns. For instance, crowdsourced data such as Google Map reviews and social media content have been utilized to analyze public perceptions of children’s activities in urban parks, offering a passive and scalable alternative to traditional surveys [42]. This approach has enabled the identification of key themes such as safety, amenities, and recreational opportunities, which vary across neighborhoods with differing demographic characteristics. Similarly, large-scale smartphone mobility data have been used to examine seasonal variations in park visitation across dozens of urban parks, revealing how environmental and infrastructural attributes interact with climate and socioeconomic conditions to influence usage patterns [43]. Furthermore, the integration of dynamic demographic data derived from mobile phone signals has advanced the spatial optimization of urban park locations, with a particular focus on addressing environmental justice and ensuring equitable access to green spaces [44]. These studies underscore the value of mobile and location-based data in capturing real-time behavioral patterns and informing more responsive, data-driven approaches to urban green space planning. While smartphone-based location data offer the clear advantage of quantitative insights into users’ spatial behavior, technical and ethical constraints hinder data acquisition. Due to these limitations, there is a relative paucity of studies that utilize smartphone-based location data to link individual exposure to green space in their daily routes to their physical and mental health compared to those that utilize self-reported surveys. This study aims to fill this gap by focusing on human interactions with green spaces using smartphone-based location data.

By analyzing the patterns of green space visits and integrating them with personal health traits and perceptions, we aim to increase understanding of the relationship between green space exposure and health within the context of individual travel. This extends the scope of existing research and contributes to the identification of correlations and effects of human interactions with green spaces. These findings are expected to serve as an important basis for policy on urban planning and public health promotion.

There are four research questions we aim to address in this study. First, can green space data and smartphone-based location data be used to effectively analyze citizen green space visitation patterns? Second, what are the associations between sociodemographic characteristics and green space visitation patterns? Third, what are the associations between green space visitation patterns and individuals’ physical and mental health status? Fourth, what is the relationship between green space visitation patterns and individuals’ perceptions of green spaces?

To this end, this study uses a combination of smartphone-based location data, green space data, and survey data to quantitatively analyze individuals’ green space visitation patterns and explore how these visitation patterns are associated with sociodemographic characteristics and with individuals’ health status and perceptions of green space.

## 2 Materials and methods

### 2.1 Ethical consideration

This study (2024-11-063) was exempted from review by the Ethics Committee of Sungkyunkwan University, and all procedures related to data collection and processing were supervised by the Data Protection Department.

### 2.2 Study area

The study area is the Seoul metropolitan area including Seoul, Incheon, and Gyeonggi provinces. This metropolitan area is the political, economic, and cultural center of South Korea, centered around the capital city of Seoul. It is very densely populated and is home to about half of the country’s population. The major parks and green spaces of Seoul play an important role in the city’s recreation and health promotion. Incheon is a major port city with green spaces and parks along its coastline. Gyeonggi-do, in addition to being the residential center of the metropolitan area, has extensive forests and national parks, providing nature access outside of the city center.

### 2.3 Workflow

**Fig 1:**
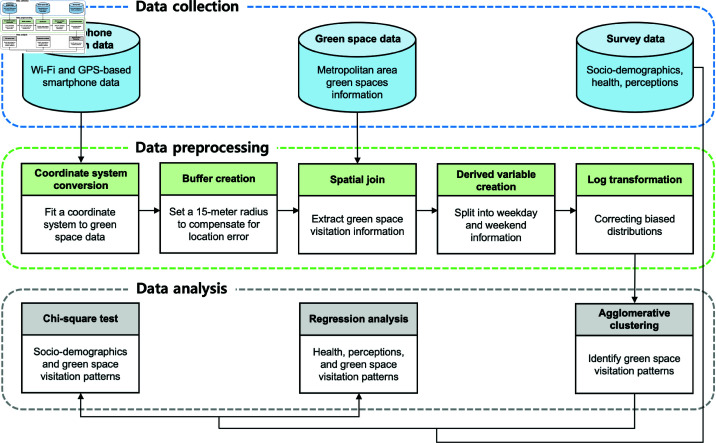
Workflows for data collection, preprocessing, and analysis.

Fig 1 shows the comprehensive workflow adopted in this study to investigate green space visitation patterns and their associations. This workflow consists of three main steps.

The first step is collection of smartphone location data, survey data, and green space data to form a multifaceted data set.

The second step, data preprocessing, involves essential transformations to ensure data compatibility and readiness for analysis. The main preprocessing steps are converting smartphone location data into a planar coordinate system and creating a buffer on the smartphone location data. The buffered smartphone location data are then spatially joined with the green space data to retain only information about individual visits to green space. Furthermore, the date information is utilized to separate green space visits into weekday and weekend variables. Finally, we apply a log transformation to normalize the distribution of the resulting derived variables to increase the robustness of the subsequent statistical analysis.

The third step of data analysis is the processing of data to identify visitation patterns through agglomerative clustering. With the identified visitation patterns and survey information, we use chi-square tests to identify associations between sociodemographic characteristics and green space visitation patterns. We also analyze the relationships between these patterns and survey responses through regression analysis to understand the associations between green space visitation and individual health and perceptions of green space.

### 2.4 Data collection

#### 2.4.1 Smartphone location data.

This study utilizes smartphone location data provided by Embrain (https://embrain.com/kor/), a leading research company specializing in consumer panels and big data analytics. The smartphone location data used in this study were collected based on WiFi signal strength and supplemented with GPS to improve the accuracy of movement detection. Therefore, these signals reflect each individual’s actual movement and visited locations, not aggregated signal coverage or infrastructure. The data were collected between January 1, 2024, and May 31, 2024, and were based on a panel of 3,421 people living in the Seoul metropolitan area of South Korea. The smartphone location data consisted of eight variables: panel_id, reg_date, duration, place_name, place_tag, place_lat, place_lng, and place_address. The panel_id is an anonymized identifier that can uniquely identify the panels , which were de-identified to protect the privacy of participants, while reg_date shows when a panel visited a specific place, consisting of date and time information (year-month-day, hour:minute:second). The duration variable is the length of time between the panel entering and leaving a specific WiFi signal range, measured in seconds. place_name is the name of the business visited by the panel, while place_tag provides information such as the specific branch or store of a particular business. place_lat and place_lng represent the latitude and longitude of the visited location, respectively, and place_address is the physical address.

#### 2.4.2 Survey data.

The survey for this study was conducted in June 2024 by Embrain. It was based on a panel of 1,715 people living in the Seoul metropolitan area of South Korea, who were all included in the smartphone location data of 3,421 people. These 1,715 individuals had previously agreed to receive survey invitations from Embrain. Informed consent for data use had already been obtained by Embrain, and the data provided to the researchers were fully anonymized.The survey consisted of three sections with 16 questions covering each of demographics, health status, and perceptions of green space. The survey was administered in Korean, and the full questionnaire is available in Supporting Information S1 Appendix.

Respondents’ demographic information consisted of gender, age, marital status, and educational background, while mental and physical health status was assessed using the EuroQol Group’s EQ-5D-5L scale [45]. The EQ-5D-5L is a tool that assesses health status in five domains: mobility, self-care, daily activities, pain/discomfort, and anxiety/depression, with five-level responses ranging from 1 (no problem) to 5 (very serious problem) in each domain. Participants selected the level of each dimension that best described their mental and physical health status, and these numbers were indexed and used in the analysis based on the EQ-5D-5L utility weight estimation equation for Korean adults [46]. The total score calculated from the EQ-5D-5L ranges from 0 (death) to 1 (perfect health) and assesses the overall mental and physical health status of the respondent.

The green space perception questionnaire used in this study was adapted from a previous study [47]. The questionnaire was divided into two parts: frequently visited green spaces and perceptions of green spaces in the neighborhood. Perceptions of frequently visited green spaces were assessed based on accessibility, comfort, maintenance, visits by all age groups, and visits by ethnic group. Each item was answered on a six-point scale from 0 (completely disagree) to 5 (completely agree). The combined score for the five items ranged from 0 to 25. Perceptions of green spaces in the neighborhood were assessed using the two criteria of safety and accessibility and exercise possibilities, each of which were also scored on a six-point scale ranging from 0 (completely disagree) to 5 (completely agree). The combined score for these two items ranged from 0 to 10. Table 1 shows the sociodemographic information of the panelists who participated in the survey.

**Table 1:** Socio-demographic information.

1-4 Variables	Categories	n	Percent (%)
Gender	Female	905	52.8
	Male	810	47.2
Age (years)	20-29	105	6.1
	30-39	405	23.6
	40-49	662	38.6
	50-59	425	24.8
	≥60	118	6.9
Marital Status	Married	1115	65.0
	Single	600	35.0
Educational Background	High school	313	18.2
	University	1234	72.0
	Graduate school	168	9.8
Total		1715	100.0

#### 2.4.3 Green space data.

This study utilized green space data from the Seoul metropolitan area as provided by MAPSEE (http://mapsee.kr/), a GIS society in the Department of Geography, College of Science, Kyung Hee University. The dataset used in this study was published on September 30, 2022. and consists of 11,902 green spaces located in the Seoul metropolitan area of South Korea, categorized by four variables: UCB, Shape_Leng, Shape_Area, and geometry.

UCB is a unique identifier assigned to each green space based on type: natural grassland, artificial grassland, coniferous forest, broadleaf forest, and mixed forest. Shape_Leng is a numerical value that quantifies the length of the boundary of each green space, providing a quantitative representation of the perimeter of the space. Shape_Area quantifies the area of each green space, indicating the ground size of the space. The geometry represents each green space as coordinate data in the form of a polygon, and the coordinate system used is Korean 1985 Modified Korea Central Belt, which is a plane coordinate system based on the Bessel 1841 ellipsoid and Transverse Mercator projection. This coordinate system allows precise representation of geographic information in the metropolitan area of South Korea to the nearest meter. It is commonly referred to by the international standard code EPSG:5174, which is a unique identification number for the coordinate reference system maintained by the European Petroleum Survey Group (EPSG) [48].

#### 2.4.4 Data accessibility.

The smartphone location data and survey data used in this study were obtained from a commercially established dataset owned by Embrain, which is subject to third-party disclosure restrictions under a contractual agreement. These data were made available for research purposes only and cannot be publicly shared. However, data access requests can be made through the following link [49]. The green space data, on the other hand, is publicly accessible and can be used without restrictions. It can be downloaded from the following link [50]

### 2.5 Data preprocessing

#### 2.5.1 Coordinate system conversion.

This study utilized the Python programming language (version 3.11.8) for data preprocessing and analysis (https://www.python.org/) and was executed in the Google Colab environment (https://colab.research.google.com/). This allowed for efficient preprocessing and analysis of large-scale data. In this study, to extract green space visitation information, it is necessary to match the longitude and latitude information of smartphone location data with the coordinate system of green space data. In this process, the longitude and latitude information of smartphone location data was stored in a geographic coordinate system according to the international standard code EPSG:4326 and converted into point objects [51]. EPSG:4326 is a WGS 84 ellipsoid-based geographic coordinate system that represents locations in terms of longitude and latitude [52]. However, due to its angular representation, it has limitations in calculating distance and area [53], and the distance between longitudes varies with latitude, even for the same longitude [54].

For green space visitation information that requires accurate distance and area calculations, it is necessary to convert EPSG:4326 data to a plane coordinate system. EPSG:5174, which is used to build green space maps for the Korean metropolitan area, uses a meter-based plane coordinate system to enable consistent calculations [53] and is suitable for calculating distances and areas in Korea.

While it is possible to convert longitude and latitude information from smartphone location data to EPSG:5174 at an early stage in the process of extracting green space visitation information, it is more common to store the raw data of longitude-latitude coordinates according to EPSG:4326 and then convert them to EPSG:5174 at a later stage when distance- and area-based analysis is required. This approach has the advantage of increasing the accuracy of distance and area calculations while maintaining the longitude and latitude information.

#### 2.5.2 Buffer creation.

The smartphone location data used in this study were collected based on WiFi signal strength, with a GPS sensor supplemented to improve the accuracy of movement detection. While WiFi-based location data can provide high accuracy indoors and in dense urban areas, it is subject to error due to signal strength and the surrounding environment. Existing studies have shown that WiFi-based location systems have an average error range of about 13-40 meters, which can vary depending on access point density and signal environment [55]. GPS devices provide higher accuracy in outdoor environments, and consumer GPS receivers have an average error of about 5 meters under ideal conditions but can be off by up to 15 meters in urban areas or environments with many obstacles [56–58].

To compensate for this error, we set a buffer of a 15-meter radius around the point objects converted to the EPSG:5174 coordinate system. The 15-meter radius was chosen based on a combination of the effective range of the WiFi signal and the maximum error of the GPS data and was used as a criterion for evaluating intersections with green spaces.

#### 2.5.3 Spatial join.

We performed a spatial join using the buffered smartphone location data and green space data. In this process, we checked for intersections in each panel’s travel route to a green space, and only the data with confirmed intersections were used in the analysis. The spatial join technique was used to assess green space visitation based on the interaction between panelists’ travel routes and green spaces.

**Fig 2:**
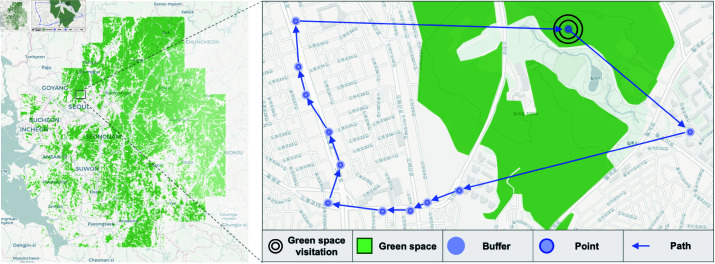
Visualization of panel locations and green space visits with a GPS buffer overlay.

Fig 2 is a visual representation of the green spaces in the Korean metropolitan area provided by MAPSEE and the daily travel behavior of a specific panel of people. The location of the panel is indicated by a blue dot, and the translucent blue circular area surrounding the dot represents the 15-meter radius buffer. Green spaces are represented by green polygons, and the path of travel of the panel is represented by blue arrows. In addition, the areas highlighted by double circles represent visits to green spaces, the extracted data of which are the focus of this study. The background map was generated using Contextily with tiles provided by CARTO, based on OpenStreetMap data. Base map and data from OpenStreetMap and OpenStreetMap Foundation.

These visuals clearly show whether and when the panelists visited green spaces along their travel routes. The data extracted through spatial joins were utilized to calculate the duration, visit count, and visit days of each panel’s daily green space visits.

#### 2.5.4 Derived variable creation.

The daily green space usage data for each panel were summed into weekdays and weekends. Daily green space visitation duration (duration), number of visits (exp_count), and number of days (exp_days) were calculated by weekdays and weekends to create six variables (weekday green space visitation duration, weekend green space visitation duration, weekday visitation count, weekend visitation count, weekday visitation days, and weekend visitation days). This distinction allowed better understanding of the differences in green space use patterns between weekdays and weekends.

#### 2.5.5 Log transformation.

**Fig 3:**
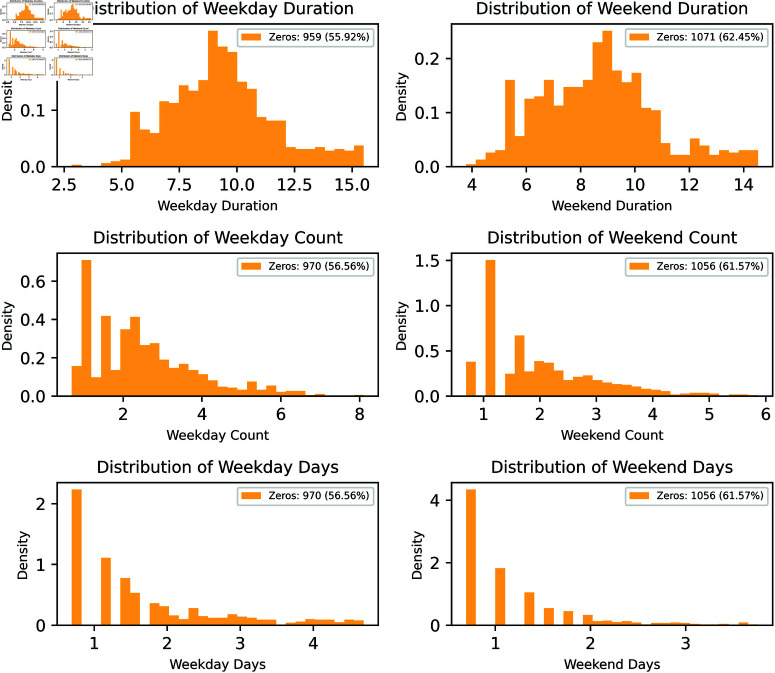
Distribution of green space visit variables after logarithmic transformation.

Skewed distributions were found in six of the calculated variables. This phenomenon can cause asymmetry between variables and affect the results of the analysis. To compensate for this, we applied a log transformation, as shown in Fig 3, to mitigate the impact of extreme values, normalize the distribution, and increasing reliability during statistical analysis and modeling. Therefore, in this study, log-transformed variables were used to analyze the green space use patterns in each panel.

### 2.6 Data analysis

#### 2.6.1 Optimal clustering approach.

In this study, we compare clustering techniques using six green space use variables to analyze the green space use patterns of the entire panel and use the Silhouette Score to establish the optimal number of clusters for each technique. The Silhouette Score is a measure of the coherence of clusters, with values ranging from -1 to 1, with higher values indicating better clustering. The clustering techniques used in the analysis were Agglomerative Clustering, DBSCAN (Density-Based Spatial Clustering of Applications with Noise), GMM (Gaussian Mixture Model), HDBSCAN (Hierarchical Density-Based Spatial Clustering of Applications with Noise), and KMeans. For Agglomerative Clustering, GMM, and KMeans, the number of clusters was set from 2 to 10 to select the optimal number of clusters based on the highest silhouette score. DBSCAN and HDBSCAN, on the other hand, do not specify the number of clusters in advance, and clusters are formed automatically based on the density of the data. We evaluated the optimal clustering performance by adjusting the eps value for DBSCAN and the min_samples value for HDBSCAN.

Table 2 shows the optimal silhouette coefficients for each method. The Agglomerative Clustering technique showed the highest silhouette coefficient and was selected as the best clustering model. The silhouette coefficient of 0.7511 for the selected Agglomerative Clustering technique is the coefficient when the data are classified into four groups.

**Table 2:** Comparison of silhouette scores by clustering algorithm.

Clustering Algorithm	Number of Clusters	eps	min_samples	Silhouette Score
Agglomerative Clustering	4	N/A	N/A	**0.7511**
DBSCAN	N/A	0.5	5	0.5074
GMM	4	N/A	N/A	0.6996
HDBSCAN	N/A	N/A	5	0.5646
KMeans	4	N/A	N/A	0.7489

Significant silhouette scores are emphasized in bold for clarity and comparison.

#### 2.6.2 Agglomerative clustering.

Agglomerative clustering is a type of hierarchical clustering that uses a bottom-up approach to form clusters by incrementally merging individual data points [59]. The formation of clusters in this technique depends on the distance measure and linkage criterion. In this study, clustering was performed using the Ward linkage criterion, which minimizes the variance between clusters. The Ward linkage criterion is designed to minimize the Sum of Squared Errors (SSE) within clusters during the clustering process and is an appropriate method for forming balanced clusters with evenly distributed data. In the Ward linkage criterion, the linkage cost of clusters A and B is calculated using (Eq 1).

$$d(A,B)=\frac{\left|A|+|B\right|}{\left|A|\cdot |B\right|}\|\mu_{A}-\mu_{B}{\|}^{2}$$ (1)

where *d*(*A*,*B*) denotes the linkage cost between clusters *A* and *B*, and |A| and |B| denote the number of data points in clusters *A* and *B*, respectively. $\mu_{A}$ and $\mu_{B}$ represent the centroids (mean) of clusters *A* and *B*, respectively, and $\|\mu_{A}-\mu_{B}{\|}^{2}$ represents the square of the Euclidean distance between the centroids of clusters *A* and *B*. This formula shows how, when merging two clusters, the pair that results in the smallest increase in the Sum of Squared Errors (SSE) within the clusters is selected and merged. By applying the Ward linkage criterion, the clustering process can maximize the internal cohesion of each cluster and optimize the separation between clusters.

In this study, the optimal number of clusters was selected based on the silhouette coefficient, and green space visits were analyzed in four groups. Six variables were used to compare the characteristics of green space visits in each group: weekday green space visit time, weekend green space visit time, number of weekday visits, number of weekend visits, number of weekday days, and number of weekend days.

#### 2.6.3 Chi-square test for differences in sociodemographic characteristics between groups.

In this study, a chi-square test was performed to determine if there was a difference in the distribution of sociodemographic characteristics between the groups. The chi-square test was calculated as shown in (Eq 2). Here, *O*_*ij*_ denotes the observed frequency of the *i*-th row and *j*-th column, *E*_*ij*_ denotes the expected frequency of the *i*-th row and *j*-th column, *r* denotes the number of rows, and *c* denotes the number of columns.

$$\chi^{2}=\sum_{i=1}^{r}\sum_{j=1}^{c}\frac{(O_{ij}-E_{ij}{)}^{2}}{E_{ij}}$$ (2)

The expected frequency is calculated as in (Eq 3), where *R*_*i*_ is the sum of the *i*-th row, and *C*_*j*_ is the sum of the *j*-th column. *N* is the total sample size.

$$E_{ij}=\frac{R_{i}\cdot C_{j}}{N}$$ (3)

Based on the chi-square test results, standardized residuals were calculated to more specifically analyze the differences in the distribution of sociodemographic characteristics between groups. The standardized residuals were calculated as shown in (Eq 4), where *R*_*ij*_ is the residual corresponding to the *i*-th row and *j*-th column. The rest of the symbols are the same as in the chi-square test formula.

$$R_{ij}=\frac{O_{ij}-E_{ij}}{\sqrt{E_{ij}}}$$ (4)

#### 2.6.4 Regression analysis of group differences in EQ-5D-5L and green recognition.

To assess the associations between green space visitation patterns and individuals’ health and green space perceptions, we conducted a regression analysis. In the first step, we built a basic model as shown in (Eq 5), with EQ-5D-5L scores, perception scores for frequently visited green spaces, and perception scores for green spaces in the neighborhood as dependent variables and green space visitation pattern information as the independent variable.

$$Y_{i}=\beta_{0}+\beta_{1}\cdot {\text{group}}_{i}+\epsilon_{i}$$ (5)

To increase the reliability of the subsequent analysis, an extended regression model was constructed by adding gender, age, marital status, and educational background as covariates, as shown in (Eq 6).

$$Y_{i}=\beta_{0}+\beta_{1}\cdot {\text{group}}_{i}+\beta_{2}\cdot {\text{gender}}_{i}\;\;+\beta_{3}\cdot {\text{age}}_{i}+\beta_{4}\cdot {\text{marital\_status}}_{i}\;\;+\beta_{5}\cdot {\text{educational\_background}}_{i}+\epsilon_{i}$$ (6)

## 3 Results

### 3.1 Characteristics of green space visitation patterns

**Fig 4:**
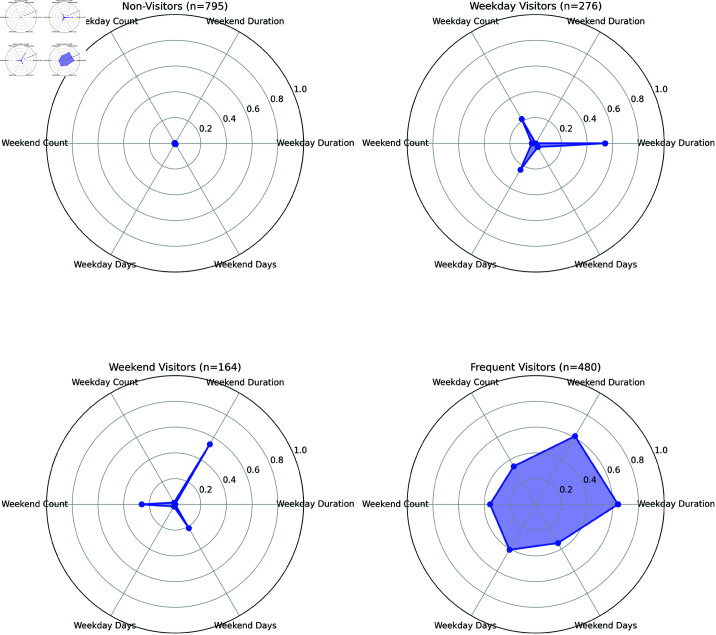
Comparison of green space visit patterns by group.

The characteristics of the four groups formed through agglomerative clustering were examined through visualization. In Fig 4, all variables were normalized to a range between 0 and 1 by applying MinMaxScaler to the preprocessed data to reduce scale differences across variables and enhance comparability between groups. Radar charts were then created using the mean values of each group to clearly visualize the characteristics of green space visitation patterns across groups. This was done for visualization purposes only, and the preprocessed data were used for analysis. Upon examining the group characteristics, the participants were categorized into four groups: 795 individuals (46.36%) who rarely visit green spaces, 276 individuals (16.09%) who primarily visit green spaces on weekdays, 164 individuals (9.56%) who mainly visit on weekends, and 480 individuals (27.99%) who frequently visit green spaces on both weekdays and weekends. Based on these characteristics, the groups were labeled as ’Non-Visitors’, ’Weekday Visitors’, ’Weekend Visitors’, and ’Frequent Visitors’, respectively, and these labels were used to interpret the patterns and characteristics of each cluster.

The analysis included clustered groups and surveys based on 1715 green space visits, with 795 survey responses from the Non-Visitors group, 276 from the Weekday Visitors group, 164 from the Weekend Visitors group, and 480 from the Frequent Visitors group. The asymmetric distribution between groups suggests that confidence intervals for results in certain groups are wider, suggesting caution in interpretation. The sociodemographic characteristics of participants by group are presented in Table 3. Each visitor group exhibits distinct demographic characteristics. The Non-Visitors group includes the largest number of individuals (46.36%) and has the lowest proportion of those with graduate-level education (6.6%). In contrast, the Weekday Visitors group is the only group in which the proportion of males (54.3%) exceeds that of females, and it also shows the highest percentage of individuals aged 60 and above (9.8%). The Weekend Visitors group, while comprising the smallest number of individuals (9.56%), has the highest proportion of married individuals (67.1%). Finally, the Frequent Visitors group stands out for having the highest percentage of individuals in their 30s (27.1%), the lowest proportion of high school graduates (13.0%), and the highest proportion of those with graduate-level education (13.5%). These patterns suggest that green space visitation behaviors may be influenced by specific socio-demographic characteristics.

**Table 3:** Socio-demographic information across visitor groups.

1-6 Variables	Categories	Non-Visitors	Weekday Visitors	Weekend Visitors	Frequent Visitors
Gender	Female	431(54.2%)	126(45.7%)	89(54.3%)	259(54.0%)
	Male	364(45.8%)	150(54.3%)	75(45.7%)	221(46.0%)
Age (years)	20-29	44(5.5%)	11(4.0%)	12(7.3%)	38(7.9%)
	30-39	185(23.3%)	57(20.7%)	33(20.1%)	130(27.1%)
	40-49	325(40.9%)	100(36.2%)	64(39.0%)	173(36.0%)
	50-59	186(23.4%)	81(29.3%)	46(28.1%)	112(23.3%)
	≥60	55(6.9%)	27(9.8%)	9(5.5%)	27(5.6%)
Marital Status	Married	513(64.5%)	178(64.5%)	110(67.1%)	314(65.4%)
	Single	282(35.5%)	98(35.5%)	54(32.9%)	166(34.6%)
Educational Background	High school	161(20.3%)	60(21.7%)	30(18.3%)	62(13.0%)
	University	581(73.1%)	185(67.0%)	115(70.1%)	353(73.5%)
	Graduate school	53(6.6%)	31(11.3%)	19(11.6%)	65(13.5%)
Total		795	276	164	480

### 3.2 Associations between sociodemographic characteristics and green space visitation patterns

In order to compare the demographic distributions among the four groups (Non-Visitors, Weekday Visitors, Weekend Visitors, and Frequent Visitors), chi-square tests and standardized residual analyses were conducted. Table 4 presents the results of the chi-square tests. The analysis revealed statistically significant differences among the groups in terms of age (*p* = 0.050)and educational background (*p*<0.001). In contrast, no significant differences were observed with respect to gender or marital status, suggesting that age and educational background are key factors differentiating the characteristics of each group.

**Table 4:** Chi-square test results for demographic variables across groups.

Variables	Chi-Square ($\chi^{2}$)	p-value	Degrees of Freedom (dof)
Gender	6.6951	0.082	3
Age (years)	21.0581	0.050	12
Marital Status	0.4553	0.929	3
Educational Background	28.3219	$<0.001^{*}$	6

Significance levels: ${\;}^{*}p\;<\;0.05$, ${\;}^{**}p\;<\;0.01$, ${\;}^{***}p\;<\;0.001$

Table 5 summarizes the standardized residual analyses for the age and educational background variables, both of which yielded significant chi-square results. According to the residual analysis, there were no statistically significant differences in age distributions across the four groups. Although the chi-square test indicated an overall significance for age, no individual category contributed disproportionately to the result. This finding suggests that the distribution of age may be relatively balanced across the groups or that minor deviations across multiple age brackets collectively accounted for the observed significance. In other words, while an overall difference in age distribution exists among the groups, it is not driven by a specific age range. In contrast, a clear difference was observed in the educational background variable in both the chi-square and residual analyses. Notably, the number of graduate degree holders in the Non-Visitors group was significantly lower than expected (|r|>2.58). Conversely, in the Frequent Visitors group, graduate degree holders were significantly over-represented, while high school graduates were significantly under-represented (|r|>2.58). These findings indicate a distinct association between educational background and green space visitation patterns.

**Table 5:** Residual analysis for age groups and educational background across groups.

1-6 Variables	Categories	Non-Visitors	Weekday Visitors	Weekend Visitors	Frequent Visitors
Age (years)	20-29	-0.6698	-1.4347	0.6182	1.5886
	30-39	-0.2000	-1.0129	-0.9205	1.5636
	40-49	1.0346	-0.6333	0.0873	-0.9023
	50-59	-0.7845	1.5239	0.8405	-0.6372
	≥60	0.0406	1.8380	-0.6799	-1.0486
Educational Background	High school	1.3205	1.3565	0.0125	**-2.7355**
	University	0.3750	-0.9644	-0.2764	0.4102
	Graduate school	**-2.8190**	0.7622	0.7321	**2.6220**

Bolded values indicate |r|>2.58, corresponding approximately to *p*<0.01.

### 3.3 Associations between physical and mental health and green space visitation patterns

The association between individuals’ physical and mental health and their patterns of green space visits can be confirmed through both the univariate model, which includes only green space visitation patterns, and the model adjusted for sociodemographic variables. Table 6 presents the results of the univariate analysis. The analysis compared all groups against the Non-Visitors group, which rarely visits green spaces. The results showed that the Weekday Visitors, Weekend Visitors, and Frequent Visitors groups had higher scores on the EQ-5D-5L scale, which measures mental and physical health. Notably, only the Weekend Visitors group(those who visit green spaces on weekends) showed a statistically significant improvement in health scores compared to the Non-Visitors group (β=0.021;p<0.01).

**Table 6:** Univariate regression analysis results for physical and mental health and green space perceptions across groups.

Greenspace measure	Coefficients	95% CI	p-value
**“ Association of green space visitation patterns with health”**			
Intercept	0.8316	0.826, 0.838	-
Group[T.Weekday Visitors]	0.0077	-0.004, 0.019	0.195
Group[T.Weekend Visitors]	0.0210	0.007, 0.035	0.004${\;}^{**}$
Group[T.Frequent Visitors]	0.0059	-0.004, 0.016	0.228
**“ Association of green space visitation patterns with perceptions of green spaces (residential area)”**			
Intercept	8.2692	8.121, 8.418	-
Group[T.Weekday Visitors]	0.1366	-0.156, 0.429	0.360
Group[T.Weekend Visitors]	0.4503	0.091, 0.810	0.014${\;}^{*}$
Group[T.Frequent Visitors]	0.3850	0.143, 0.627	0.002${\;}^{**}$
**“ Association of green space visitation patterns with perceptions of green spaces (frequently visited)”**			
Intercept	19.9333	19.624, 20.242	-
Group[T.Weekday Visitors]	0.5304	-0.079, 1.139	0.088
Group[T.Weekend Visitors]	1.2801	0.532, 2.028	0.001${\;}^{**}$
Group[T.Frequent Visitors]	1.0187	0.515, 1.523	$<0.001^{***}$

Significance levels: ${\;}^{*}p<0.05$, ${\;}^{**}p<0.01$, ${\;}^{***}p<0.001$

S2 Appendix shows the analysis after adjusting for sociodemographic variables, and the differences observed between groups in the univariate model remained consistent even after adjustment (β=0.020;p<0.01). This suggests that the timing of green space visits may play a more critical role in promoting health than the frequency or duration of visits. On the other hand, the Weekday Visitors and Frequent Visitors groups (those who visit both on weekdays and weekends) did not show significant differences compared to the Non-Visitors group.

Among the control variables, marital status and educational background were also significantly associated with mental and physical health. Specifically, individuals who were single reported lower health scores than those who were married, and those with only a high school education showed significantly lower health outcomes compared to those with graduate-level education. While these findings are not the main focus of this study, they suggest that sociodemographic characteristics also play a meaningful role in shaping health outcomes and merit further investigation in future research.

### 3.4 Associations between green space perceptions and visitation patterns

The association between individuals’ perceptions of green spaces and their green space visitation patterns was evident in both the univariate model(which included only green space visitation patterns) and the model adjusted for sociodemographic characteristics. Table 6 presents the results of the univariate analyses, in which all visitation groups were compared against the Non-Visitors group. In contrast to the findings related to mental and physical health, both the Weekend Visitors and Frequent Visitors groups showed significantly higher perceptions of green spaces than the Non-Visitors group. Specifically, the Weekend Visitors group (β=0.450;p<0.05) and the Frequent Visitors group (β=0.385;p<0.01) reported significantly higher perceptions of green spaces within their residential neighborhoods. Additionally, both the Weekend Visitors group (β=1.280;p<0.01) and the Frequent Visitors group (β=1.018;p<0.001) demonstrated markedly higher perceptions of the green spaces they frequently visit, compared to the Non-Visitors group.

S3 Appendix presents the results after adjusting for sociodemographic characteristics, including gender, age, marital status, and educational background. The group differences observed in the univariate model remained consistent even after these adjustments. The Weekend Visitors group (β=0.418;p<0.05) and the Frequent Visitors group (β=0.352;p<0.01) continued to show significantly higher perceptions of neighborhood green spaces than the Non-Visitors group. Similarly, the perception of green spaces frequently visited remained significantly higher for the Weekend Visitors group (β=1.221;p<0.01) and the Frequent Visitors group (β=0.955;p<0.001). These findings suggest that the frequency and context of green space visits play a critical role in shaping individuals’ perceptions of these environments. In contrast, the Weekday Visitors group did not show any statistically significant differences in green space perception compared to the Non-Visitors group.

Among the control variables, age, marital status, and educational background also showed significant associations with perceptions of green spaces. In particular, older adults (age ≥60) tended to report more positive perceptions than younger groups. Additionally, single individuals and those with lower educational attainment (e.g., high school or university education only) reported significantly lower perception scores compared to their counterparts. While these variables are not the central focus of this study, these results suggest that sociodemographic factors also play a meaningful role in shaping how green spaces are perceived. Future research could further explore these interactions in greater depth.

## 4 Discussion

This study identified green space visitation patterns among smartphone users residing in the Seoul metropolitan area of South Korea who consented to data collection and comprehensively analyzed how these patterns are associated with sociodemographic characteristics, individual health status, and perceptions of green spaces. Previous studies on green space visitation have primarily relied on self-reported surveys [47, 60], which are subject to recall bias, exaggeration, and subjective distortion, thus limiting their ability to objectively capture actual visitation frequency or patterns [27, 28]. To address these limitations, recent studies have increasingly utilized smartphone-based location data to quantify green space usage behaviors [39], analyze visitation characteristics [40], and explore the selection and use of urban green spaces [41]. While smartphone-based location data offer a clear advantage in quantifying users’ spatial behaviors, technical and ethical constraints make data acquisition and application challenging. As a result, studies linking green space exposure within individuals’ mobility patterns to physical and mental health outcomes remain relatively limited compared to self-reported studies. By integrating smartphone-based location data with survey responses and green space geospatial data, this study seeks to bridge the gap with existing self-report-based research and to provide a multi-dimensional analysis of human interaction with green space. In particular, this study distinguishes itself from previous smartphone location-based research by applying a unique methodological framework and topic focus.

Green space visitation patterns were identified and categorized into four groups: Non-Visitors, Weekday Visitors, Weekend Visitors, and Frequent Visitors, based on the presence and timing of green space use. Each group’s characteristics were then analyzed in relation to health status and perceptions of green spaces. The analysis revealed that green space visitation behavior varies not only based on frequency but also by timing and individual life patterns. This finding supports previous claims that simply measuring visitation frequency fails to capture the complexity of human–environment interactions [61]. Instead, the timing of visits should be considered when designing urban green space strategies. The segmentation into four visitation groups highlights the need for differentiated management strategies such as time-specific demand forecasting, tailored programming, and improved accessibility. Differences in the distribution of sociodemographic characteristics across groups indicate a meaningful association between educational background and green space use. The Non-Visitors group had a significantly lower number of graduate school graduates than expected, while the Frequent Visitors group had a higher-than-expected number of graduate school graduates and a significantly lower number of high school graduates. This finding supports existing research suggesting that higher educational attainment is positively associated with green space use [62]. These disparities underscore the need for environmental education programs and public campaigns to promote awareness of environmental benefits and encourage equitable use of green spaces, which may help reduce environmental inequalities. The analysis of associations between visitation patterns and physical/mental health outcomes indicated that the timing of visits had a greater influence than visitation frequency. The Weekend Visitors group demonstrated significantly better mental and physical health scores than the Non-Visitors group. This difference is consistent with previous studies indicating that people prefer visiting large parks with well-equipped picnic facilities on weekends, when they have more leisure time [63]. These findings suggest that weekend leisure activities in green spaces can contribute meaningfully to personal health, highlighting the need for urban planning that provides accessible and desirable green spaces for weekend use. Conversely, no significant differences in health status were observed between the Weekday Visitors and Frequent Visitors groups, suggesting that weekday visits may often occur as part of routine daily activities (such as commuting to work or school), and thus may offer limited benefits for rest and recovery. In terms of perceptions, both the Weekend Visitors and Frequent Visitors groups exhibited significantly higher levels of positive perceptions toward green spaces than the Non-Visitors group. This suggests that perceived benefits of green space do not necessarily align with health outcomes and that visitation experience plays a distinct role in shaping perception [64]. The asymmetry between health and perception outcomes implies that green space visitation exerts multidimensional and complex effects. Accordingly, beyond improving physical accessibility, strategies that enhance the quality of green space experiences (such as time-tailored programs, community-based activities, and emotionally satisfying environmental design) should be promoted.

This study emphasizes the need to move beyond a purely quantitative approach to urban greening. Instead, effective planning must consider sociodemographic traits, life patterns, and users’ diverse needs. The identification of four distinct visitation groups (Non-Visitors, Weekday Visitors, Weekend Visitors, and Frequent Visitors) further underscores the importance of differentiated management strategies. Tailored approaches, such as improving accessibility for Non-Visitors, offering flexible weekday programs, and expanding weekend-oriented green spaces, can help meet the unique needs of each group. The observed differences in visitation patterns by educational background indicate that green space usage is closely tied to cultural and educational environments. At the community level, integrating environmental education programs, cultural events, and local festivals into green space use could enhance the perceived value and accessibility of these spaces and promote more equitable utilization across social groups. Furthermore, the significantly better mental and physical health observed among weekend green space visitors suggests the value of creating “weekend-specialized green spaces” with concentrated amenities such as large parks, recreational facilities, and playgrounds, to support leisure time with family and friends. The more positive perceptions among those who visited on weekends or both weekdays and weekends indicate that direct experience strongly shapes green space awareness. Therefore, interactive and engaging content (such as AR/VR experiences and participatory events) should be considered to foster a virtuous cycle between visitation and perception. This integrated approach may serve as a meaningful strategy to achieve four key goals: differentiated green space management, reduction of environmental inequality, promotion of public health, and enhancement of green space awareness.

This study has several limitations. As a cross-sectional study, it is not suited to inferring causal relationships. Since visitation patterns may change over time, the effects of such changes on health and perceptions could not be assessed. A longitudinal research design is recommended for future studies to better understand the causal pathways and long-term impacts of green space exposure. In this study, location data were collected from January 1 to May 31, 2024, and survey data on health and perceptions were collected in June 2024. Future studies could incorporate additional data from June 1 to December 31, 2024, along with follow-up surveys in January 2025. This would allow researchers to examine whether individuals’ visitation patterns changed over time and how those changes were associated with health outcomes and perceptions. For example, mixed-effects modeling could be used to examine whether individuals classified as Non-Visitors from January to May who became Frequent Visitors from June to December experienced improvements in health and perceptions. This approach would enable a more nuanced understanding of the potential causal links and long-term effects of green space exposure. Lastly, as this study focused on residents in the Seoul metropolitan area, the generalizability of the results to other urban or rural contexts is limited. Future research should incorporate comparative analyses across diverse geographic regions to enhance the external validity of the findings.

## 5 Conclusion

This study integrated smartphone-based location data, survey responses, and green space geospatial data to categorize green space visitation patterns among residents of the Seoul metropolitan area. Based on this analysis, green space use behaviors were found to vary not only by frequency but also by the timing of visits and individuals’ daily life patterns. Accordingly, users were classified into four groups: Non-Visitors, Weekday Visitors, Weekend Visitors, and Frequent Visitors. These findings highlight the need for differentiated management strategies, such as time-specific demand forecasting, improving accessibility, and developing tailored programs that address usage patterns concentrated on specific days or times. Notably, the differences in educational background across groups indicate that green space use is closely associated with access to educational and cultural resources. This suggests that expanding accessibility through environmental education programs and culturally relevant activities should be considered as key policy initiatives. Furthermore, the Weekend Visitors group demonstrated the highest levels of both mental and physical health, and both the Weekend and Frequent Visitor groups showed significantly more positive perceptions of green spaces compared to the Non-Visitor group. This implies that green space visitation affects not only health outcomes but also the formation of positive perceptions. In this regard, initiatives such as developing “weekend-specialized green spaces,” as well as incorporating digital content or local festivals, may simultaneously enhance health benefits and perceptual engagement. These results emphasize the importance of going beyond the mere physical expansion of green spaces and instead underscore the need for customized green space planning and management strategies that reflect variations in life patterns, socioeconomic backgrounds, and temporal demand.

However, this study is based on a cross-sectional design, which limits the ability to draw causal inferences. Moreover, as the analysis focused on residents of the Seoul metropolitan area, there are limitations to the generalizability of the findings. Future research should incorporate longitudinal data and comparative analyses across diverse regions to more precisely identify the long-term impacts of green space use on health outcomes and public perception. Such research can provide a more nuanced and robust foundation for the development of effective policies in urban planning, public health, and environmental management.

## Supporting information

S1 Appendix Questionnaire(KR/EN). (DOCX)

S2 Appendix Corrected univariate regression analysis results for physical and mental health across groups. (DOCX)

S3 Appendix Corrected univariate regression analysis results for green space perceptions across groups. (DOCX)
