# Supplementary material for: Associations of green space visitation patterns with sociodemographics, health, and perceptions: A cluster analysis using smartphone Wi-Fi and GPS data
Source: PLoS One. 2025 Jun 27;20(6):e0325697. doi: 10.1371/journal.pone.0325697 (PMC12204507; doi:10.1371/journal.pone.0325697)
Supplement: S1 Appendix — (DOCX) [file pone.0325697.s001.docx]

**Questionnaire(KR)**

**Part I. 인구통계**

1. 귀하의 성별은 무엇입니까?
   - 여성
   - 남성
2. 귀하의 연령대는 어디에 해당합니까?
   - 20–29세
   - 30-39세
   - 40-49세
   - 50-59세
   - 60세 이상
3. 귀하의 결혼 상태는 무엇입니까?
   - 기혼
   - 미혼
4. 귀하의 최종 학력은 무엇입니까?
   - 고등학교 졸업
   - 대학교 졸업
   - 대학원 졸업

**Part II. 건강 관련 삶의 질**

문항 5-9는 EuroQol Group에서 개발한 EQ-5D-5L 도구를 사용하여 건강 관련 삶의 질을 측정하였습니다. 저작권 제한으로 인해 설문 문항 전체는 본문에 포함되지 않았으며, 자세한 내용은 [https://euroqol.org](https://euroqol.org/)를 참조하시기 바랍니다.

**Part III. 녹지에 대한 인식(빈번한 방문)**

1. 자주 방문하는 녹지 공간은 접근성이 좋다고 생각하십니까?
   - 전혀 그렇지 않다
   - 그렇지 않다
   - 약간 그렇지 않다
   - 약간 그렇다
   - 그렇다
   - 매우 그렇다
2. 자주 방문하는 녹지 공간은 잘 관리되어 있다고 생각하십니까?
   - 전혀 그렇지 않다
   - 그렇지 않다
   - 약간 그렇지 않다
   - 약간 그렇다
   - 그렇다
   - 매우 그렇다
3. 자주 방문하는 녹지 공간은 다양한 연령대의 사람들이 자주 이용한다고 생각하십니까?
   - 전혀 그렇지 않다
   - 그렇지 않다
   - 약간 그렇지 않다
   - 약간 그렇다
   - 그렇다
   - 매우 그렇다
4. 자주 방문하는 녹지 공간은 쾌적한 분위기를 제공한다고 생각하십니까?
   - 전혀 그렇지 않다
   - 그렇지 않다
   - 약간 그렇지 않다
   - 약간 그렇다
   - 그렇다
   - 매우 그렇다
5. 자주 방문하는 녹지 공간은 다양한 인종의 사람들이 이용한다고 생각하십니까?
   - 전혀 그렇지 않다
   - 그렇지 않다
   - 약간 그렇지 않다
   - 약간 그렇다
   - 그렇다
   - 매우 그렇다

**Part IV. 녹지에 대한 인식(주거지 인근)**

1. 귀하의 주거지 인근에서 안전하고 접근 가능한 쾌적한 녹지를 쉽게 찾을 수 있다고 생각하십니까?
   - 전혀 그렇지 않다
   - 그렇지 않다
   - 약간 그렇지 않다
   - 약간 그렇다
   - 그렇다
   - 매우 그렇다
2. 귀하의 주거지 인근에서 운동할 수 있는 녹지를 쉽게 찾을 수 있다고 생각하십니까?
   - 전혀 그렇지 않다
   - 그렇지 않다
   - 약간 그렇지 않다
   - 약간 그렇다
   - 그렇다
   - 매우 그렇다

**Questionnaire(EN)**

**Part I. Demographics**

1. What is your gender?
   - Female
   - Male
2. Which age group do you belong to?
   - 20–29 years
   - 30-39 years
   - 40-49 years
   - 50-59 years
   - 60 years or older
3. What is your marital status?
   - Married
   - Single
4. What is your highest level of education completed?
   - High school graduate
   - University graduate
   - Graduate school graduate

**Part II. Health-Related Quality of Life**

Questions 5–9 use the EQ-5D-5L instrument developed by the EuroQol Group to measure health-related quality of life. Due to copyright restrictions, the full questionnaire items are not included in this document. For detailed information, please visit [https://euroqol.org](https://euroqol.org/).

**Part III. Perceptions of Green Spaces (Frequent Visits)**

1. Do you think the green space you frequently visit is easily accessible?
   - Strongly disagree
   - Disagree
   - Slightly disagree
   - Slightly agree
   - Agree
   - Strongly agree
2. Do you think the green space you frequently visit is well maintained?
   - Strongly disagree
   - Disagree
   - Slightly disagree
   - Slightly agree
   - Agree
   - Strongly agree
3. Do you think the green space you frequently visit is used by people of various age groups?
   - Strongly disagree
   - Disagree
   - Slightly disagree
   - Slightly agree
   - Agree
   - Strongly agree
4. Do you think the green space you frequently visit provides a pleasant atmosphere?
   - Strongly disagree
   - Disagree
   - Slightly disagree
   - Slightly agree
   - Agree
   - Strongly agree
5. Do you think the green space you frequently visit is used by people from diverse racial or ethnic backgrounds?
   - Strongly disagree
   - Disagree
   - Slightly disagree
   - Slightly agree
   - Agree
   - Strongly agree

**Part IV. Perceptions of Green Spaces (Near Residence)**

1. Do you think it is easy to find safe, accessible, and pleasant green spaces near your residence?
   - Strongly disagree
   - Disagree
   - Slightly disagree
   - Slightly agree
   - Agree
   - Strongly agree
2. Do you think it is easy to find green spaces for physical activity near your residence?
   - Strongly disagree
   - Disagree
   - Slightly disagree
   - Slightly agree
   - Agree
   - Strongly agree
