# Supplementary material for: Associations of green space visitation patterns with sociodemographics, health, and perceptions: A cluster analysis using smartphone Wi-Fi and GPS data
Source: PLoS One. 2025 Jun 27;20(6):e0325697. doi: 10.1371/journal.pone.0325697 (PMC12204507; doi:10.1371/journal.pone.0325697)
Supplement: S2 Appendix — (DOCX) [file pone.0325697.s002.docx]

**S2 Appendix. Corrected univariate regression analysis results for physical and mental health across groups.**

| **Greenspace measure** | **Coefficients** | **95% CI** | **p-value** |
| --- | --- | --- | --- |
| **”Association of green space visitation patterns with health”** | | | |
| Intercept | 0.8383 | 0.815, 0.862 | - |
| Group[T.Weekday Visitors] | 0.0076 | -0.004, 0.019 | 0.198 |
| Group[T.Weekend Visitors] | 0.0201 | 0.006, 0.034 | 0.005** |
| Group[T.Frequent Visitors] | 0.0038 | -0.006, 0.013 | 0.432 |
| Gender[T.Male] | -0.0058 | -0.014, 0.002 | 0.160 |
| Age[T.30-39] | 0.0103 | -0.008, 0.029 | 0.281 |
| Age[T.40-49] | 0.0087 | -0.010, 0.027 | 0.363 |
| Age[T.50-59] | 0.0133 | -0.006, 0.033 | 0.181 |
| Age[T.>=60] | 0.0221 | -0.002, 0.046 | 0.068 |
| Marital Status[T.Single] | -0.0119 | -0.021, -0.003 | 0.012* |
| Education Background[T.High school] | -0.0316 | -0.048, -0.015 | < 0.001*** |
| Education Background[T.University] | -0.0054 | -0.019, 0.008 | 0.443 |

Significance levels: *p < 0.05, **p < 0.01, ***p < 0.001
