# Supplementary material for: Associations of green space visitation patterns with sociodemographics, health, and perceptions: A cluster analysis using smartphone Wi-Fi and GPS data
Source: PLoS One. 2025 Jun 27;20(6):e0325697. doi: 10.1371/journal.pone.0325697 (PMC12204507; doi:10.1371/journal.pone.0325697)
Supplement: S3 Appendix — (DOCX) [file pone.0325697.s003.docx]

**S3 Appendix. Corrected univariate regression analysis results for green space perceptions across groups.**

| **Greenspace measure** | **Coefficients** | **95% CI** | **p-value** |
| --- | --- | --- | --- |
| **”Association of green space visitation patterns with perceptions of green spaces (residential area)”** | | | |
| Intercept | 8.5669 | 7.975, 9.159 | - |
| Group[T.Weekday Visitors] | 0.0908 | -0.199, 0.381 | 0.540 |
| Group[T.Weekend Visitors] | 0.4184 | 0.063, 0.774 | 0.021* |
| Group[T.Frequent Visitors] | 0.3528 | 0.111, 0.594 | 0.004** |
| Gender[T.Male] | -0.0998 | -0.305, 0.105 | 0.340 |
| Age[T.30-39] | 0.1673 | -0.302, 0.637 | 0.485 |
| Age[T.40-49] | 0.1045 | -0.366, 0.575 | 0.663 |
| Age[T.50-59] | 0.4449 | -0.046, 0.936 | 0.076 |
| Age[T.>=60] | 0.9238 | 0.326, 1.522 | 0.002** |
| Marital Status[T.Single] | -0.4466 | -0.681, -0.212 | < 0.001*** |
| Education Background[T.High school] | -0.5568 | -0.963, -0.151 | 0.007** |
| Education Background[T.University] | -0.3155 | -0.659, 0.028 | 0.072 |
| **”Association of green space visitation patterns with perceptions of green spaces (frequently visited)”** | | | |
| Intercept | 20.4441 | 19.215, 21.674 | - |
| Group[T.Weekday Visitors] | 0.4349 | -0.168, 1.038 | 0.157 |
| Group[T.Weekend Visitors] | 1.2218 | 0.484, 1.960 | 0.001** |
| Group[T.Frequent Visitors] | 0.9557 | 0.455, 1.457 | < 0.001*** |
| Gender[T.Male] | -0.2392 | -0.665, 0.186 | 0.270 |
| Age[T.30-39] | 0.6856 | -0.290, 1.661 | 0.168 |
| Age[T.40-49] | 0.5806 | -0.397, 1.558 | 0.244 |
| Age[T.50-59] | 1.0657 | 0.046, 2.085 | 0.041* |
| Age[T.>=60] | 2.3173 | 1.076, 3.559 | < 0.001*** |
| Marital Status[T.Single] | -0.9916 | -1.478, -0.505 | < 0.001*** |
| Education Background[T.High school] | -1.2434 | -2.087, -0.400 | 0.004** |
| Education Background[T.University] | -0.8268 | -1.541, -0.113 | 0.023* |

Significance levels: *p < 0.05, **p < 0.01, ***p < 0.001
